# Detection of Aortic Arch Calcification in Apolipoprotein E‐Null Mice Using Carbon Nanotube–Based Micro‐CT System

**DOI:** 10.1161/JAHA.112.003358

**Published:** 2013-02-22

**Authors:** John M. S. Wait, Hirofumi Tomita, Laurel M. Burk, Jianping Lu, Otto Z. Zhou, Nobuyo Maeda, Yueh Z. Lee

**Affiliations:** 1 Department of Physics and Astronomy, University of North Carolina at Chapel Hill, Chapel Hill, NC (J.S.W., L.M.B., J.L., O.Z.Z., Y.Z.L.); 2 Department of Pathology and Laboratory Medicine, University of North Carolina at Chapel Hill, Chapel Hill, NC (H.T., N.M.); 3 Curriculum in Applied Science and Engineering, University of North Carolina at Chapel Hill, Chapel Hill, NC (O.Z.Z.); 4 Department of Radiology, University of North Carolina at Chapel Hill, Chapel Hill, NC (Y.Z.L.)

**Keywords:** aortic arch atherosclerosis, calcification, carbon nanotube, micro‐CT, mouse strain

## Abstract

**Background:**

We performed in vivo micro‐computed tomography (micro‐CT) imaging using a novel carbon nanotube (CNT)–based x‐ray source to detect calcification in the aortic arch of apolipoprotein E (apoE)–null mice.

**Methods and Results:**

We measured calcification volume of aortic arch plaques using CNT‐based micro‐CT in 16‐ to 18‐month‐old males on 129S6/SvEvTac and C57BL/6J genetic backgrounds (129‐apoE KO and B6‐apoE KO). Cardiac and respiratory gated images were acquired in each mouse under anesthesia. Images obtained using a CNT micro‐CT had less motion blur and better spatial resolution for aortic calcification than those using conventional micro‐CT, evaluated by edge sharpness (slope of the normalized attenuation units, 1.6±0.3 versus 0.8±0.2) and contrast‐to‐noise ratio of the calcifications (118±34 versus 10±2); both *P*<0.05, n=6. Calcification volume in the arch inner curvature was 4 times bigger in the 129‐apoE KO than in the B6‐apoE KO mice (0.90±0.18 versus 0.22±0.10 mm^3^, *P*<0.01, n=7 and 5, respectively), whereas plaque areas in the inner curvature measured in dissected aorta were only twice as great in the 129‐apoE KO than in the B6‐apoE KO mice (6.1±0.6 versus 3.7±0.4 mm^2^, *P*<0.05). Consistent with this, histological calcification area in the plaques was significantly higher in the 129‐apoE KO than in the B6‐apoE KO mice (16.9±2.0 versus 9.6±0.8%, *P*<0.05, 3 animals for each).

**Conclusions:**

A novel CNT‐based micro‐CT is a useful tool to evaluate vascular calcifications in living mice. Quantification from acquired images suggests higher susceptibility to calcification of the aortic arch plaques in 129‐apoE KO than in B6‐apoE KO mice.

## Introduction

Micro‐computed tomography (micro‐CT) based on thermionic x‐ray sources has been commonly used for visualizing anatomic structures of mice. However, the limited temporal resolution and high‐frequency, nonperiodic cardiac and respiratory motion of mice make it difficult to image moving structures. We recently developed novel x‐ray sources based on carbon nanotubes (CNTs), which provide electrons through room temperature field emission.^1–2^ CNT‐based micro‐CT allows us to generate short x‐ray pulses and control the x‐ray exposure precisely, providing higher temporal and spatial resolution sufficient to visualize the moving structures of living mice.^3^

Aortic arch calcification has been shown to be independently associated with an increased risk of cardiovascular diseases.^4^ We previously demonstrated that apolipoprotein E (apoE)–null mice with a 129S6/SvEvTac strain background (129‐apoE KO) develop more atherosclerotic plaques in the aortic arch than those with a C57BL/6J background (B6‐apoE KO).^5–7^ In this study, we explored the feasibility of detecting calcification in aortic arch plaques using CNT‐based micro‐CT and compared aortic arch calcification volume between the 2 strains of apoE‐knockout (KO) mice.

## Methods

### Mice

Two apoE‐null mouse strains, 129‐apoE KO (129/SvEvTac inbred) and B6‐apoE KO (>10 generations' backcross to C57BL/6J), of old male mice (16 to 18 months old) were maintained on normal chow and bred in our mouse facility.^5,8^ All experiments were carried out under protocols approved by the Institutional Animal Care and Use Committee of the University of North Carolina at Chapel Hill.

### Carbon Nanotube–Based Micro‐CT

**Figure 1.**
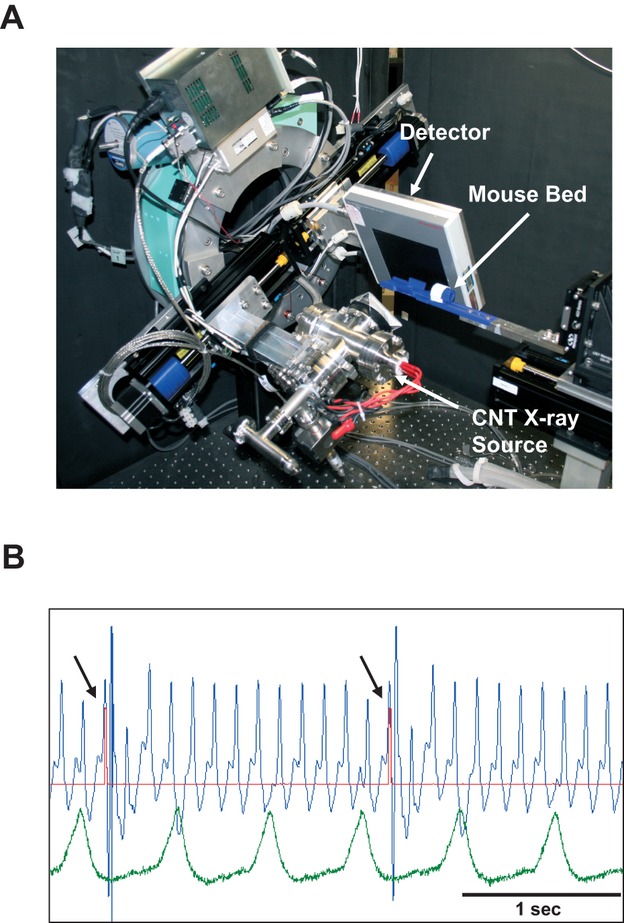
A, Custom‐built carbon nanotube micro–computed tomography (micro‐CT) assembly. B, Representative cardiac trace (upper, blue signals) and respiratory trace (below, green signals). The x‐ray pulses (red signals, arrows) are shown at the end‐diastolic cardiac phase on the R wave and at the end‐expiration phase of respiration. There is noise immediately after the x‐ray pulse. CNT indicates carbon nanotube.

The micro‐CT scanner used in this study was built in‐house and based on a carbon nanotube cathode field emission x‐ray source (Figure 1A).^1–3^ The x‐ray source in this scanner has a cold cathode that may be switched on and off electronically in much less than a microsecond, allowing high‐flux pulses of 15 ms or shorter temporal duration and 3‐mA cathode current, suitable for prospective cardiac gating. The scanner was operated in step‐and‐shoot mode, with 400 projections acquired over 200 degrees of gantry rotation and at a 50‐kVp anode voltage with an external filtration of 0.5 mm aluminum. The average imaging time for each mouse was between 15 and 25 minutes. The entrance dose was measured previously and found to be 0.20 Gy.^2^ After acquisition, images were reconstructed using a filtered back‐projection algorithm with COBRA (Exxim) commercial software to a 77‐μm isotropic voxel size in DICOM format at 512×512 per image.

### Image Acquisition

Mice were anesthetized with an initial dose of 2.5% isoflurane and sustained at 1% to 1.5% without intubation, adjusted to maintain consistent respiration for the duration of imaging. If contrast was utilized, immediately prior to imaging, via the tail vein, mice were administered a bolus of the commercial iodinated blood pool contrast agent Fenestra VC (ART Advanced Research Technologies) at the recommended dose of 0.1 mL/10 g animal body weight. Respiration motion was tracked using a small pressure sensor placed beneath each subject's abdomen while lying in the prone position on a custom‐built ABS plastic bed inside the CT scanner. Cardiac phase was tracked with electrocardiogram sensors placed on 3 of the animal's paws. Physiological information was tracked using BioVet software (m2m Imaging), and image acquisition was prospectively gated to organ motion so that x‐ray projection images were acquired only during a preset desired phase of both cardiac and respiratory cycles. Respiratory and cardiac gating effectively eliminated motion blur from the resulting images without additional radiation dose to the subject. In this study, images were acquired during the end‐expiration phase of respiration with the end‐diastolic cardiac phase on the R wave (Figure 1B).

### Conventional Micro‐CT

Imaging on a conventional micro‐CT with a traditional thermionic source was also performed using a CT 120 (Gamma Medica Inc) according to the manufacturer's protocols. The imaging protocol consisted of cardiac and respiratory gating performed at 80 kV and 32 mA, with a 16‐ms exposure over 220 views. Imaging time with this system was ≈10 minutes.

### Comparison Between CNT‐Based Micro‐CT and Conventional Micro‐CT

Two apoE‐KO mice, one of each genotype, were scanned for direct comparison of CNT‐based micro‐CT and conventional micro‐CT using the above protocols. After reconstruction, the DICOM images were transferred for offline analysis using ImageJ (NIH, Bethesda MD). The most inferior cluster of identifiable calcifications was selected. A line profile was drawn across the calcification cluster using ImageJ to measure the sharpness of the calcification edge. A region of interest in the soft tissues was used to provide a baseline value for the profiles. The HU values were normalized to the mean values of nonmoving bone in the slice of interest. The slope from the peak to the nearest point at baseline was calculated. Contrast to noise estimates was also measured, normalized by the number of imaging projections.

### Calcification Quantification

Calcification was quantified using a custom‐made MATLAB program (The Mathworks Inc). Rectangular volumes containing the aortic arch and the 3 great vessels (innominate artery, left carotid artery, and left subclavian artery) were identified. A threshold was then derived from a region of interest drawn in uncalcified myocardial tissue to represent soft tissue, and another region of interest was drawn to represent bone. The program examined each image and selected pixels representing calcification using a threshold defined by a mean radiodensity +4 standard deviations. As the program scanned the VOI corresponding to each structure, each slice containing calcifications was displayed alongside a binary image showing which pixels were identified as calcifications, so the user could identify errors. After the program finished scanning through the image stack, a composite image was displayed of all the calcifications identified in the user‐defined aortic branch artery region. The user then selected rectangular VOIs corresponding to the innominate, left subclavian, and left carotid arteries based on the distribution of calcifications. Finally, the program displayed the number of voxels identified as calcifications in each branch artery and the entire heart, the threshold radiodensity used to define calcifications, and the radiodensity of the region of bone selected as a scaling factor. Calcification volume is reported as volume not corrected for density.

### Aortic Arch Measurement

After obtaining images using CNT‐based micro‐CT, mice were anesthetized with 2.5% avertin and then perfused with 4% paraformaldehyde under physiological pressures. The aortic tree was dissected free of surrounding tissue under a dissection microscope. The aortic samples were then placed in a flat transparent chamber 1.2 mm in depth, and their images were captured. Plaque areas in the aortic arch were measured using ImageJ software as previously described.^6–7^ Cross‐sectional histological preparations of the aortic arch between the innominate artery and left carotid artery were made at 50‐μm intervals, and the mean cross‐sectional plaque size was determined from 3 cross‐sections. To detect plaques, the sections were stained by Sudan IV and counterstained with hematoxylin. Calcification of the plaques was also detected by von Kossa stainings. The plaque and calcification areas were then measured using ImageJ software by hand, and their ratio was calculated.

### Statistics

All data are expressed as mean±SEM. Differences between 2 groups were compared with the Mann‐Whitney *U* test. Repeated‐measures analysis was performed for comparison of calcification volume in the 3 major branches. Differences were considered significant at *P*<0.05.

## Results

### Comparison Between CNT‐Based Micro‐CT and Conventional Micro‐CT

**Figure 2.**
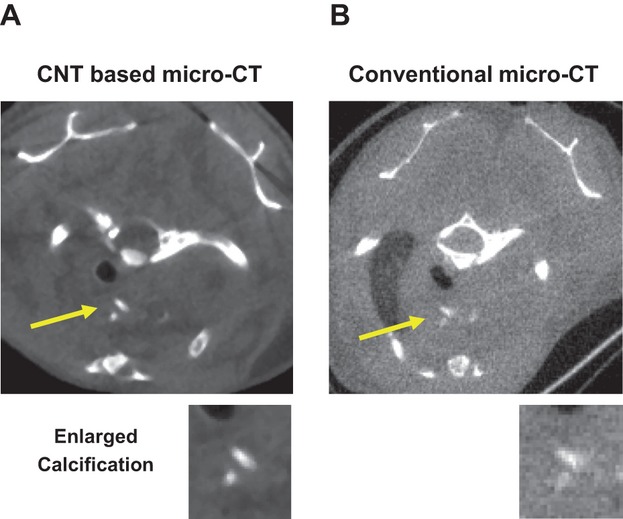
Cardiac and respiratory gated micro‐CT images from CNT‐based micro‐CT (A) and conventional micro‐CT (B) using the same living mouse. A cluster of the calcifications are shown in the middle of the thorax (yellow arrows). Enlarged calcification images are also shown. The CNT micro‐CT images demonstrate increased sharpness of the calcifications compared with the conventional micro‐CT. CT indicates computed tomography; CNT, carbon nanotube.

Imaging on a CNT‐based micro‐CT and a conventional micro‐CT was performed using the same living mouse. The sharpness of the edge of the calcifications, as measured by the slope of the normalized attenuation units, was approximately double for the CNT micro‐CT compared with the conventional micro‐CT (1.6±0.3 versus 0.8±0.2, *P*<0.05, n=6, 3 sets of each genotype). This was matched by the contrast to noise value of the calcifications (118±34 versus 10±2, *P*<0.05, n=6, 3 sets of each genotype). These indicate that the arch calcifications were both qualitatively and quantitatively more distinct on the gated CNT micro‐CT system. Representative images from the 2 devices of the arch calcifications are shown in Figure 2.

### Calcification in the Plaques

**Figure 3.**
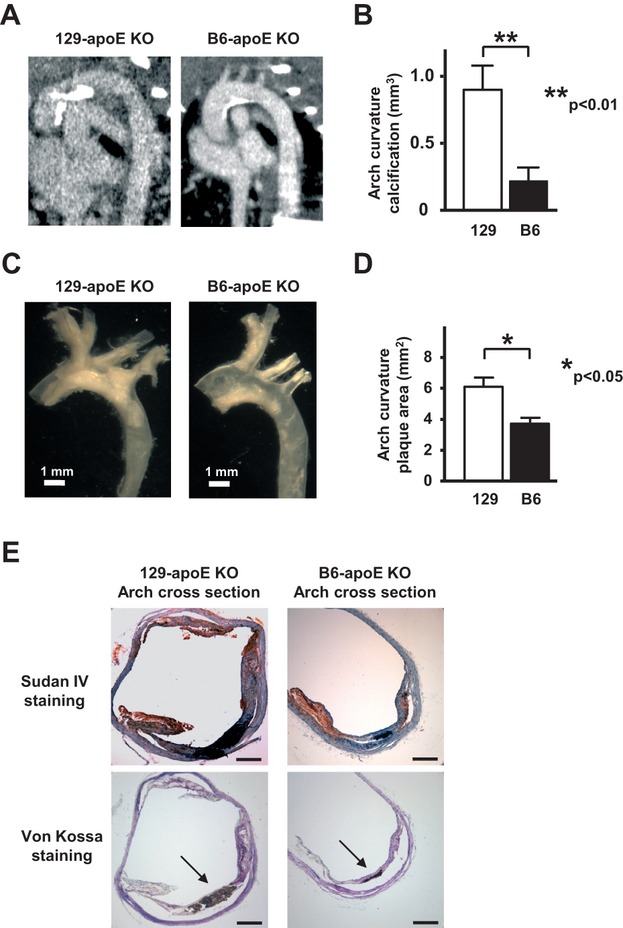
A, Representative carbon nanotube micro‐CT images of 129‐apoE KO and B6‐apoE KO mice. White areas at the inner curvature of the aortic arch indicate calcifications. B, Calcification volume in the aortic arch of the 2 strains. C, Representative images of excised aortas. D, Comparison between the 2 strains of plaque areas in the aortic arch. E, Representative arch plaques by cross‐section stained with Sudan IV and counterstained with hematoxylin. Arch calcification was detected by von Kossa staining (brown, arrows). Scale bar, 200 μm. CT indicates computed tomography; KO, knockout.

Seven 129‐apoE KO and 5 B6‐apoE KO mice were imaged, and all mice survived the imaging procedure. Representative aortic arch images of the 129‐apoE KO and B6‐apoE KO mice using the CNT‐based micro‐CT are shown in Figure 3A, in which calcifications in the inner curvature are clearly detected. Calcification volume in the inner curvature was 4 times larger in the 129‐apoE KO mice than in the B6‐apoE KO mice (0.90±0.18 versus 0.22±0.10 mm^3^, *P*<0.01, n=7 and 5, respectively; Figure 3B), whereas the plaque areas in the inner curvature of the aortic arch measured using captured light images were only twice greater in the 129‐apoE KO than in the B6‐apoE KO mice (6.1±0.6 versus 3.7±0.4 mm^2^, *P*<0.05, n=7 and 5, respectively; Figure 3C and 3D). These indicate that the percentage of calcified plaque in the atherosclerotic plaques was greater in the 129‐apoE KO than in the B6‐apoE KO mice. In contrast, there were no significant differences between the 2 groups in calcification volume in the arch branches (Table 1).

**Table 1.** Comparison of Calcification Volume in the 3 Major Branches Between the 129‐apoE KO (n=7) and B6‐apoE KO (n=5) Mice Evaluated by CNT Based Micro‐CT

	129‐apoE KO	B6‐apoE KO
Innominate artery (mm^3^)	0.17±0.06	0.15±0.08
Left carotid artery (mm^3^)	0.13±0.08	0.03±0.02
Left subclavian artery (mm^3^)	0.07±0.07	0.00002±0.00002

KO indicates knockout; CNT, carbon nanotype; CT, computed tomography. *P*=0.17 for genotype, *P*=0.08 for branch artery, and *P*=0.79 for interaction.

### Histological Assessment

Representative cross‐sectional arch plaques of the 129‐apoE KO and B6‐apoE KO mice stained by Sudan IV are shown in Figure 3E. Calcification area in the plaques was also detected by von Kossa staining in the 2 groups. The histological calcification area divided by the plaque area was significantly higher in the 129‐apoE KO than in the B6‐apoE KO mice (16.9±2.0 versus 9.6±0.8%, *P*<0.05, 3 animals for each genotype).

## Discussion

In this study using a newly developed CNT‐based micro‐CT with cardiac and respiratory gating, we have successfully quantified calcification in the aortic arch plaques of living mice. CNT‐based sources are able to achieve a 10 ms or better temporal resolution, not directly achievable using conventional thermionic sources. As a result, we were able to gate cardiac and respiratory motion in a straightforward manner, allowing simplified animal handling without the need for intubation.

Heart contraction causes movement of the vascular wall and thereby motion blur in the acquired image.^9^ In addition, because the R‐wave interval at 600 beats per minute is 100 ms, errors of pulse control >5 to 10 ms could result in significant additional blur of structures. One of the key advantages of our CNT‐based micro‐CT system is the ability to control the x‐ray pulses to at least a microsecond level. Although in vivo imaging of arch calcification has already been reported in B6‐apoE KO mice using a conventional micro‐CT scanner,^10–11^ our data clearly show that images using the CNT‐based micro‐CT had better spatial resolution for aortic plaque calcification than those using a conventional micro‐CT scanner, as demonstrated by significantly improved sharpness of the calcifications and the higher contrast to noise of the calcifications on the CNT‐based micro‐CT. The dramatic difference in the contrast‐to‐noise value of the calcifications between the 2 micro‐CT scanners may be primarily a result of the blur during acquisition.

Strain‐dependent difference in arterial wall calcification has been reported, with the B6 strain most susceptible to arterial calcification.^12^ Although the underlying molecular mechanism of the strain differences in arch calcification still remain unclear, our finding that the aortic plaques of the 129‐apoE KO mice contain proportionally more calcification than the plaques of the B6‐apoE KO mice indicates that the susceptibility may also be location specific. We also note that because aortic plaques begin to develop earlier in 129‐apoE KO mice than in B6‐apoE KO mice,^5^ calcification content could reflect an aging process of the individual plaques.

One of the important limitations of the present study is that atherosclerotic plaques are not detectable by a CNT‐based micro‐CT even after the use of contrast enhancement. As more specific molecular probes for atherosclerotic plaques have been developed, these may be helpful to detect total plaques.^13–15^ In addition, a relative threshold to identify and quantify aortic calcifications did not discriminate the values between calcium and other minerals such as iron, zinc, and magnesium. Finally, lack of statistical significance in some of the comparisons in the present study may be a result of low power.

In conclusion, our novel CNT‐based micro‐CT is a useful tool to evaluate vascular calcification in living mice. Further studies regarding strain‐dependent difference in arch calcification are warranted.

## Acknowledgment

We thank Michael K. Altenburg, Svetlana Zhilicheva, and Shinja Kim for their excellent technical support.
